# Staging of Primary Abdominal Lymphomas: Comparison of Whole-Body MRI with Diffusion-Weighted Imaging and ^18^F-FDG-PET/CT

**DOI:** 10.1155/2015/104794

**Published:** 2015-12-22

**Authors:** Alessandro Stecco, Francesco Buemi, Martina Quagliozzi, Mariangela Lombardi, Alberto Santagostino, Gian Mauro Sacchetti, Alessandro Carriero

**Affiliations:** ^1^Radiology Department, “Maggiore della Carità” University Hospital, University of Eastern Piedmont, Corso Mazzini 18, 28100 Novara, Italy; ^2^Oncohaematology Department, Sant'Andrea Hospital, Corso Mario Abbiate 21, 13100 Vercelli, Italy; ^3^Nuclear Medicine Department, “Maggiore della Carità” University Hospital, University of Eastern Piedmont, Corso Mazzini 18, 28100 Novara, Italy

## Abstract

*Background*. The purpose of this study was to compare the accuracy of whole-body MRI with diffusion-weighted sequences (WB-DW-MRI) with that of ^18^F-FDG-PET/CT in the staging of patients with primary gastrointestinal lymphoma. *Methods*. This retrospective study involved 17 untreated patients with primary abdominal gastrointestinal lymphoma. All patients underwent ^18^F-FDG-PET/CT and WB-DW-MRI. Histopathology findings or at least 6 months of clinical and radiological follow-up was the gold standard. The Musshoff-modified Ann Arbor system was used for staging, and diagnostic accuracy was evaluated on a per-node basis. *Results*. WB-DW-MRI exhibited 100% sensitivity, 96.3% specificity, and 96.1% and 100% positive and negative predictive values (PPV and NPV), respectively. The sensitivity, specificity, and PPV and NPV of PET/CT were 95.9%, 100%, and 100% and 96.4%, respectively. There were no statistically significant differences between the two techniques (*p* = 0.05). The weighted kappa agreement statistics with a 95% confidence interval were 0.97 (0.95–0.99) between the two MRI readers and 0.87 (0.82–0.92) between the two methods. *Conclusions*. WB-DW-MRI appears to have a comparable diagnostic value to ^18^F-FDG-PET/CT in staging patients with gastrointestinal lymphoma.

## 1. Introduction

The gastrointestinal (GI) tract is the most common extranodal site in lymphoma, accounting for 5% to 20% of all cases [1]. Indeed, lymphomas frequently arise in the mesenteric or retroperitoneal nodes, and the abundance of lymphoid tissue in the GI tract makes this a susceptible site for secondary involvement.

Primary GI lymphomas are, however, uncommon, comprising fewer than 5% of all (GI) cancers. Indeed, according to Dawson et al. [2], a diagnosis of primary GI lymphoma should be restricted to localized disease of stages IE and IIE, whereas Lewin et al.'s [3] system requires that patients exhibit GI symptoms or a predominant lesion.

The stomach is the most common site of primary GI lymphomas, followed by the small intestine and then the ileocaecal valve [4]. The majority of such tumours (90%) are of B-cell lineage, and T-cell lymphomas and Hodgkin's lymphoma are rare. Some histological subtypes are more often found at certain locations, for example, mucosa-associated lymphoid tissue (MALT) lymphoma in the stomach, mantle cell lymphoma (MCL) in the terminal ileum, jejunum, and colon, enteropathy-associated T-cell lymphoma (EATL) in the jejunum, and follicular lymphoma (FL) in the duodenum [5]. Multifocal tumours are particularly common in MALT lymphoma and follicular lymphoma.

Accurate diagnosis and staging of primary GI lymphomas, an especially heterogeneous group of tumours, are fundamental for treatment stratification [6]. Staging of GI lymphomas is generally performed by means of Musshoff's modified version of Ann Arbor staging [7], with the international prognostic index being used to define the prognostic subgroups. However, the system is less than optimal for documenting certain features specific to primary GI lymphoma, in particular diffuse and incurable infiltration of the GI tract. Due to this deficiency, many staging protocols and reporting systems have been proposed, and among these the Paris staging system stands out due to its ability to record the depth of tumour infiltration and specific lymphoma spread, as well as the extent of nodal involvement [8].

Various procedures are employed in diagnosis and follow-up and to provide data for pretreatment staging, including endoscopic ultrasound (EUS), endoscopic biopsies, computed tomography (CT), magnetic resonance imaging (MRI), ^18^F-fluorodeoxyglucose positron emission tomography (^18^F-FDG-PET), and/or molecular markers [9]. EUS and CT are the most widely performed techniques, and CT of the chest, abdomen, and pelvis exhibits high sensitivity and specificity in staging GI lymphomas. The sensitivity and specificity of CT-based staging of FL, MCL and diffuse large B-cell lymphoma (DLBCL) can be increased even further, to 80% and 90%, respectively, by the integration of ^18^F-FDG-PET. However, this provides no added benefit for MALT lymphomas [10]. Moreover, EUS is considered superior to CT scan in terms of locoregional staging, as it provides details of visceral wall involvement, and for the detection of perivisceral adenopathies [11].

Whole-body diffusion-weighted MRI (WB-DW-MRI) has also been extensively studied as a method of staging lymphomas [9, 12], in addition to other tumours [13, 14]. Indeed, whole-body imaging techniques can be used to assess supradiaphragmatic nodal and extranodal sites, thereby providing information vital for abdominal lymphoma staging. Despite the promise being shown by WB-DW-MRI in this area, ^18^F-FDG-PET/CT remains the standard of reference [14]. Nevertheless, as WB-DW-MRI does not expose the patient to ionizing radiation, its validation as a GI lymphoma staging tool would be of great clinical significance. However, to the best of our knowledge there have been no studies investigating the role of WB-DW-MRI in the staging of this type of lymphoma to date.

We therefore set out to evaluate the performance of WB-DW-MRI in staging primary abdominal GI lymphomas with respect to the current preferred method, ^18^F-FDG-PET/CT. As Paris staging has not yet been universally accepted, we elected to use the modified Ann Arbor system, using histopathological findings to confirm the diagnoses.

## 2. Materials and Methods

### 2.1. Patients

This retrospective study involved 17 untreated patients with primary abdominal GI lymphoma (12 males and 5 females; age range: 34.8–82 years, mean age: 63.1 years) diagnosed between July 2007 and February 2015. All patients underwent WB-DW-MRI and ^18^F-FDG-PET/CT for staging purposes. Five patients also underwent EUS and nine abdominal CT scan with contrast before therapy. Diagnosis of primary GI lymphoma was confirmed histopathologically in all patients. At least 6 months of clinical and radiological follow-up was available for each patient.

According to Ann Arbor staging with Musshoff's modification, no patients were classed as stage 0 (0), two as stage I, seven as stage II, five as stage III, and three as stage IV.

All patients underwent PET/CT and MRI in the 3 weeks preceding treatment. The time interval between MRI and PET/CT scans was 0–33 days. Subsequently, 1 patient was treated by means of surgical resection and chemotherapy, while 11 received chemotherapy alone and 5* H. pylori* eradication and chemotherapy. Of the treated patients, 9 achieved complete remission, 6 achieved partial remission, and 2 showed no response. Tables 1 and 2 show the clinicopathological features of patients and the histological classification and site of origin of the GI lymphomas diagnosed.

Informed consent was obtained from all patients and the local ethical committee approved this study.

### 2.2. Whole-Body MRI

All patients underwent MRI on a superconductive 1.5 T magnet (Achieva, Philips, Best, Netherlands, release 2.5) using a q-body coil, with the patient positioned “feet first” on an extended anatomical coverage table featuring rolling-table technology (MobiTrak, Philips). The light visor was pointed at the orbitomeatal plane.

After multiplanar and multistack scout pulse sequence reconstruction into a whole-body scout, by means of proprietary software (MobiView, Philips), a STIR-EPI single-shot pulse sequence (diffusion-weighted imaging with background suppression, DWIBS) (TR/TE = 4284/68 ms; TI = 180 ms; matrix = 108*∗*67; voxel size = 5 mm; NSA = 8; thickness = 6 mm; gap =1 mm; slices = 30; FOV = 530 (RL), 341 (AP), and 180 (FH); acquisition time: 02 min 21 secs) was acquired in the axial plane. This was repeated in free breathing for up to four stacks to encompass all anatomical districts, from head to foot.

The MR protocol involved the acquisition of T2-STIR (TR/TE = 3819/165; 2 NEX; matrix = 336*∗*120; thickness = 6 mm; gap = 1 mm; slices = 47; FOV: 530 (RL), 265 (FH), and 328 (AP); acquisition time: 1 min 8 secs) and spin echo-T1 (TR/TE = 788/18; 1 NEX; matrix = 208*∗*287; thickness = 6 mm; gap = 1 mm; slices = 43; FOV: 530 (RL), 300 (AP), and 265 (FH); acquisition time: 1 min 10 secs) sequences in the sagittal and coronal planes.

### 2.3. PET-CT

PET-CT scans were taken on a hybrid Siemens (Siemens, Erlangen, Germany) system consisting of a lutetium oxyorthosilicate (LSO) PET scanner (HI-REZ) with Pico-3D electronics and a 16-row CT device (Somatom Sensation 16).

The PET component is a high-resolution scanner with a spatial resolution of 4.7 mm and has no septa, thus allowing 3-dimensional-only acquisition. Together with the PET system, the CT scanner is used both for attenuation correction of PET data and for localization of ^18^F-FDG uptake in PET images. The intravenously administrated dose of ^18^F-FDG was 3,5 mBq/Kg of body weight and imaging was performed 60 minutes after administration of the tracer.

Acquired images were reconstructed using the attenuation weighted-OSEM (ordered subset expectation maximization) iterative reconstruction, with 2 iterations, 8 subsets. Fourier rebinning was used to reduce the 3D dataset to a 2-dimensional equivalent dataset, and a 4 mm full width at half maximum Gaussian filter was applied to the image after reconstruction along the axial and transaxial directions. The data were reconstructed over a 128*∗*128 matrix with 5.3 mm pixel size and 2 mm slice thickness. Processed images were displayed in coronal, transverse, and sagittal planes.

## 3. Image Analysis

After acquisition and selection of the highest *b* value, the native axial images were reformatted on a stack-by-stack basis as a single 340 mm thick maximum intensity projection (MIP) image in the coronal plane, multiple 4 mm thick multiplanar reconstructions (MPRs) in the coronal plane, and multiple 4 mm thick MPRs in the sagittal plane, oriented to include the midline as well as the spine and paraspinal regions. All reformatted images were then fused by means of the smooth fusion algorithm in MobiView software (Philips) to obtain whole-body MIP and MPR images. The grey scale was subsequently inverted to enable viewing of abnormalities (increased signal) as grey areas of varying intensity against a white background, in a PET-like visualization window, as suggested by Takahara et al. [15].

All whole-body MIP and MPR images were saved in the scanner's image database in patient-specific files. The native axial slices were separated on the basis of the *b* value, and those acquired with *b* = 1,000 s/mm^2^ were also saved in the image database on optimal window settings. T2-STIR and SE-T1 scan data were then merged into a single whole-body image using the same method and software.

All MR and PET/CT image sets were processed by an experienced trained radiologist, anonymized, and stored in DICOM format on a CD-ROM marked with a patient- and session-specific identification code.

MR images were read independently by two experienced MRI radiologists (A.S. and A.C. with 15 and 25 years of experience in MR imaging, resp.), both blind to each other's findings and the patient's clinical status. PET/CT images were read by a specialist with 18 years of experience in nuclear medicine (G.S.).

The three readers recorded their findings on a predesigned spreadsheet (Excel, Microsoft, Redmond, USA) listing the following nodal and extranodal sites [16]:

1. Abdominal GI locations: stomach, duodenum, small bowel, colon/rectum, and multiple sites.
2. Other extranodal locations: spleen, liver, kidney, head and neck, lung, and bone.
3. Subdiaphragmatic nodal locations: paragastric, mesenteric, para-aortic, paracaval, pelvic, and inguinal.
4. Supradiaphragmatic nodal locations: laterocervical, axillary, supraclavicular, and mediastinal.

As Abdulqadhr et al. [17], the evaluation of possible nodal lesions was performed with MIP images of the whole-body DW sequences. The lesion was then confirmed by checking axial DW images using a value of *b* = 1000 mm^2^/sec. A *b* value of 0 mm^2^/sec together with *b* = 1000 mm^2^/sec was used to rule out T2 shine-through effect [18] and to get anatomical information. The axial DW images were correlated to MIP images by references lines.

Lesions identified on DWIBS were considered positive for the disease if [18]:

1. the major axis measurement was greater than 1 cm (on axial DWIBS sequences),
2. their signal intensity on DWIBS was greater than that of the spinal cord,
3. there were coalescent lymph nodes or nodal masses,
4. lymph nodes were present in regions where there are normally no lymph nodes.

Central necrosis was considered a sign of malignancy, regardless of the size of the lymph node [19].

Extranodal lesions were identified as follows:

1. Presence of areas of restricted diffusion at GI tract and parenchymal organs with respect to the background signal.
2. Correlation of the above with signal abnormalities on the morphological sequences (coronal and sagittal STIR/T1w sequences).

Bone marrow signals were considered abnormal when greater than that of muscle on T2-weighted sequences and/or when presenting a “mild” restriction of diffusion on DWIBS [20].

As apparent diffusion coefficient (ADC) values for small organs and tissues are affected by respiratory motion and fail to accurately distinguish malignant from benign lesions [21], due to partial volume effects, and since inter- and intraobserver variability afflict reproducibility of the measures [22], we neither calculated nor used ADC to characterize lesion tissues. Instead, a lesion was considered positive on ^18^F-FDG-PET/CT scans in the presence of greater focal or diffuse ^18^F-FDG uptake than background activity in a location incompatible with normal/physiology (or unrelated to physiological sites of tracer uptake) [23, 24]. The location of ^18^F-FDG uptake was always verified by CT. Lymph nodes with a shortest transverse diameter of ≥1 cm [23] or masses/coalescent lymph node were also considered positive.

The patients were staged by Ann Arbor staging with Musshoff's modification using the data yielded by each imaging technique. The two sets of results were then compared with each other and the results of biopsy or when this was not possible with at least 6 months of clinical and radiological follow-up (CT, MRI, and PET/CT), the standard of reference. A reduction in the size of the lesion after therapy was taken as evidence that the lesion was positive for lymphoma [22].

## 4. Statistical Analysis

Sensitivity, specificity, accuracy, and positive and negative predictive values were calculated for each diagnostic method on a “per-node” basis (N-staging). Analysis of the accuracy of WB-DW-MRI, ^18^F-FDG-PET/CT, and ^18^F-FDG-PET without CT in the assessment of the individual disease stage of each patient was also performed. Regarding the gastrointestinal lymphoma manifestation, we only calculated the ^18^F-FDG-PET/CT and WB-DW-MRI detection rates, as their large fields of view impede the accurate staging of locoregional tumour extension (stage I).

Cohen's *k* statistics were used to calculate the interobserver agreement between the two MR readers and between WB-DW-MRI and ^18^F-FDG-PET/CT. Agreement was defined as poor at *k* < 0.2, fair at *k* > 0.2 < 0.4, moderate at *k* > 0.4 < 0.6, good at *k* > 0.6 < 0.8, and very good at *k* > 0.8.

McNemar's test was used to determine the statistical significance of differences between WB-DW-MRI and ^18^F-FDG-PET/CT interpretations. A *p* value of <0.05 was regarded as statistically significant. MedCalc (MedCalc Software, Belgium) was used for all statistical analyses.

## 5. Results

### 5.1. Per-Node Basis

WB-DW-MRI was true-positive for 75 (100%) of the lymphomatous node groups and true-negative for 79 (96%) of the nonmetastatic node groups, while ^18^F-FDG-PET/CT was true-positive for 71 (94%) of the lymphomatous node groups and true-negative for 83 (100%) of the nonlymphomatous node groups. WB-DW-MRI exhibited 100% (CI 95%, 95.2% to 100%) sensitivity, 96.3% (CI 95%, 89.5% to 99.2%) specificity, and 96.1% (CI 95%, 89.1% to 99.2%) and 100% (CI 95.3% to 100%) positive and negative predictive values, respectively. The sensitivity, specificity, and PPV and NPV of ^18^F-FDG-PET/CT were 95.9% (CI 95%, 88.6% to 99.1%), 100% (CI 95%, 95.60 to 100%), and 100% (CI 95%, 94.9% to 100%) and 96.4% (CI 95%, 90% to 99.27%), respectively.

McNemar's test revealed no statistically significant differences between ^18^F-FDG-PET/CT and WB-DW-MRI (*p* < 0.05).

The weighted kappa statistics, with a 95% confidence interval of agreement, were 0.97 (0.95–0.99) between the two MRI readers and 0.87 (0.82–0.92) between the two methods.

### 5.2. Per-Patient Analysis and Gastrointestinal Lymphoma Detection Rate

**Figure 1:**
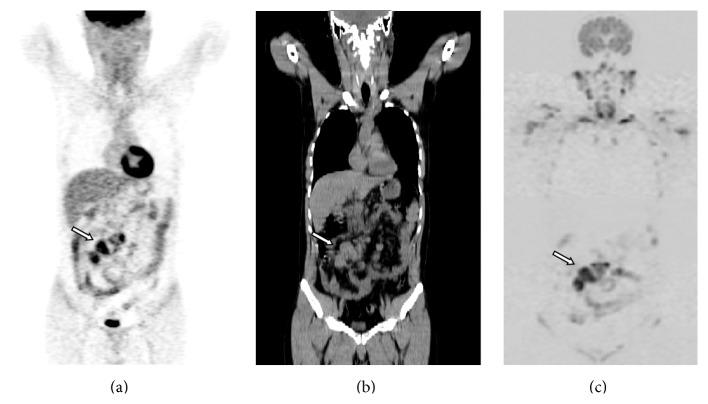
A 38-year-old man with intestinal DLBCL (diffuse large B-cell lymphoma): (a) MIP image of ^18^F-FDG-PET, (b) coronal whole-body CT, and (c) MIP image of DWI. All techniques detected the primary intestinal lesion (arrow).

**Figure 2:**
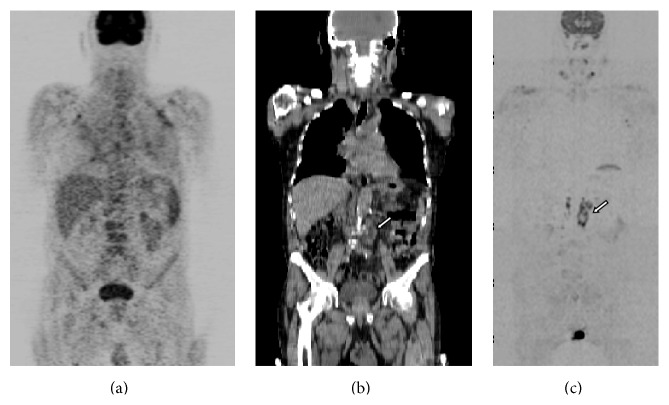
An 82-year-old man with gastric MALT lymphoma: (a) MIP image of ^18^F-FDG-PET showing no uptake in any lymph node regions, giving stage 0; (b) coronal whole-body CT, showing multiple para-aortic lymph nodes, thus giving stage II (arrow); (c) MIP image of DWI showing the same findings, as high signal intensity in the same nodal location, giving stage II (arrow). However, all techniques, even DWI, failed to find the primary gastric lesion.

Of the 17 lymphoma patients, 16 were staged the same by WB-DW-MRI and ^18^F-FDG-PET/CT (94%). Of those 16 patients, 1 was classed as stage IV, and the remaining 15 were distributed between stages 0 and III (Figures 1 and 2, Table 3). In the case in which staging differed, WB-DW-MRI classed the patient as stage IV, predicting bone marrow (BM) invasion, while ^18^F-FDG-PET/CT classed the patient as stage III. The bone marrow infiltration, and therefore the accuracy of the higher staging, provided by MRI, was confirmed by bone biopsy (Table 4).

In the 2 patients with low-grade MALT lymphoma, WB-DW-MRI and ^18^F-FDG-PET/CT agreed (both stage II), but ^18^F-FDG-PET alone predicted a far lower stage (stage 0). In the mismatched regions, enlarged lymph nodes with no F-FDG uptake were present on CT (>1 cm) (Figure 2, Table 4).

^18^F-FDG-PET/CT and WB-DW-MRI both detected the gastrointestinal lymphoma manifestation in 10 out of the 17 patients (60%), while ^18^F-FDG-PET alone detected it in only 8 of 17 (47%). In other words, WB-DW-MRI and ^18^F-FDG-PET/CT downstaged 2 patients classed as stage 1 by the gold standard, whereas ^18^F-FDG-PET alone downstaged 4 patients (Table 4).

## 6. Discussion

To our knowledge, this is the first study to evaluate the role of WB-DW-MRI in the N-staging of primary GI lymphoma. Although it was not possible for us to confirm the diagnosis in all lymph nodes by histopathology (a common problem in radiological research, as it would be highly unethical to biopsy all suspected lesions) WB-DW-MRI staging was generally in full agreement with that provided by the standard of reference. In the majority of our cases, there were no differences between WB-DW-MRI and ^18^F-FDG-PET/CT in this regard. However, both yielded a lower stage than the standard of reference in two patients with indolent stage 1 MALT lymphoma (Table 4), with neither PET/CT nor WB-DW-MRI being able to detect any morphologic abnormality or ^18^F-FDG uptake/restriction of diffusivity in the known location of the gastrointestinal lymphoma in either of these two patients. ^18^F-FDG-PET alone, on the other hand, failed to detect ^18^F-FDG uptake in these and a further two cases (total 4 patients) with indolent MALT lymphoma (both stage II), which were successfully staged by integrating CT data (Figure 2, Table 4).

As there was a high preponderance of early-stage (I-II) indolent MALT lymphoma in our group, it is perhaps unsurprising that, overall, both ^18^F-FDG-PET/CT and WB-DW-MRI had a GI lymphoma detection failure rate of 40% as compared to 53% with ^18^F-FDG-PET alone. Indeed, in 33 cases of MALT lymphoma, Perry et al. [25] showed ^18^F-FDG-PET/CT detected active disease in 100% of advanced cases (stages III-IV) but in only 42.3% of cases of early-stage disease (I-II). It seems reasonable to assume, therefore, that the early stage, small size, and low metabolism/low ^18^F-FDG uptake were behind both the downstaging of two patients and the missed gastrointestinal lymphoma manifestations.

In our study, WB-DW-MRI detected one more patient with positive BM lesion than ^18^F-FDG-PET/CT, correctly upstaging this patient with respect to ^18^F-FDG-PET/CT. This finding is in line with those by Abdulqadhr et al. [17] showing that WB-DW-MRI correctly upstaged 3 patients with small lymphocytic lymphoma/chronic lymphatic leukaemia (SLL/CLL) as compared to ^18^F-FDG-PET/CT. They postulated that the increased signal seen in SLL/CLL lesions was ascribable to the small, closely packed tumour cells causing restricted water molecule movement. However, in our two missed cases of stage 1 indolent MALT lymphoma, we failed to find any significant restriction. However, the normal focal or diffuse restriction of the small primary lesions may have been hidden by the bowel loops.

On a per-node basis, we found WB-DW-MRI to be more sensitive (100% versus 96.3%) but less specific (95.1% versus 100%) than ^18^F-FDG-PET/CT. Overall, WB-DW-MRI detected a higher number of false-positive lymph nodes at the laterocervical, axillary, and inguinal nodal locations than ^18^F-FDG-PET/CT, which were confirmed as reactive nodes in the follow-up. That being said, McNemar's test revealed no statistically significant differences between the two investigations, and the interobserver agreement was “very good” (>0.8).

This makes an interesting contribution to the growing debate regarding the excessive exposure to radiation during diagnostic procedures. Indeed, although ^18^F-FDG-PET/CT is widely used in the management of lymphomas, it involves exposing patients to a substantial dose of radiation, which is of particular concern in young adults and children [26, 27] and in patients who require repeated follow-up. A nonionizing imaging technique, such as MRI, with similar functional imaging capacity would therefore be afforded an important role in this setting, especially during follow-up.

Our results, like those of Abdulqadhr et al. [17], appear to suggest a role for WB-DW-MRI, combined with ^18^F-FDG-PET/CT, in initial staging and provided that there is agreement between the two techniques as a standalone follow-up imaging technique. This would not only be safer for patients, but also improve cost-effectiveness and total examination time, both important limitations of ^18^F-FDG-PET/CT, in the long term. Indeed, ^18^F-FDG-PET/CT costs roughly twice as much as WB-MRI and takes considerably longer (60 minutes of waiting time after ^18^F-FDG injection versus 30–35 minutes) [28].

Nevertheless, there are some limitations to our study. First and foremost, it was retrospective in nature and therefore not reliant on a standardized MRI protocol. Due to the heterogeneity of the MRI protocols adopted in our institution over the years, we only considered DWIBS and STIR/T1. However, it would have been preferable to have been able to add a WB-DW-MRI protocol with axial T1w or T2w sequences, so as to shed light on the morphological characteristics of the lymph nodes at the laterocervical, axillary, and inguinal locations, and thereby potentially eliminate false-positives. However, as no such data was available for our patients, we were unable to take them into account.

Secondly, the rarity of primary GI lymphoma meant that we were only able to consider a limited number of patients. Further studies are needed, not only to confirm our findings in a larger sample, but also to refine the staging procedure for gastric MALT lymphoma. Although indolent, this type of tumour may be multifocal [29, 30], transform to DLBCL [31], and it is difficult to diagnose, endoscopic findings being normal in the majority of cases. As multiple organs are generally involved, endoscopic biopsies usually need to be taken from multiple sites of the stomach and duodenum, encompassing both normal and abnormal regions [10, 32]. Our results indicate that this highly invasive procedure may one day be more accurately guided by DWIBS sequences, which could also prove useful by predicting the transformation of this tumour into higher-grade lymphoma (indeed, PET has already been recommended as a means of assessing the recurrence or transformation of various lymphomas [33]), although, once again, more research is necessary.

## 7. Conclusions

Our results suggest WB-DW-MRI as a promising technique for staging patients with primary GI lymphomas, and, pending the results of future studies and improvements to its performance, it may provide a radiation-free alternative to ^18^F-FDG-PET/CT.

## Figures and Tables

**Table 1:** Clinicopathological features and sites of origin of tumours investigated.

Characteristics	Gastric	Intestinal
*Mean age (yrs)*	60.2	64.5
*Gender*		
Male	3	9
Female	2	3
*Histology*		
Low-grade B-cell	5	4
High-grade B-cell	0	8
*Stage*		
IE	2	0
IIE	3	4
IIIE	0	6
IVE	0	2
*Treatment*		
Surgery alone	0	0
Nonsurgical	6	10
Both	0	1
*Response to treatment*		
Complete remission	5	4
Partial remission	0	6
No response	0	2

**Table 2:** Histological classification and sites of origin.

Histology type	Gastric	Intestinal	Total
*Low-grade B-cell*			
MALTL^*∗*^	3	5	8
Follicular lymphoma	1	4	5
*High-grade B-cell*			
DLBCL^*∗∗*^	1	3	4

^*∗*^MALTL, mucosa-associated lymphoid tissue lymphoma; ^*∗∗*^DLBCL, diffuse large B-cell lymphoma.

**Table 3:** Staging of lymphoma provided by WB-DW-MRI and ^18^F-FDG-PET/CT.

Stage	WB-DW-MRI				
	0	I	II	III	IV
^18^F-FDG-PET/CT					
0	2				
I		0			
II			7		
III				6	1
IV					1

**Table 4:** Staging by both techniques: mismatched sites behind differences in staging with respect to standard of reference.

Patient	WB-DW-MRI	^18^F-FDG-PET/CT	^18^F-FDG-PET	Mismatched regions	Gold standard
Gastric MALTL^*∗*^	0	0	0	Primary gastric lesion	Stage I. Positive EUB and endoscopy (ulcerated gastric lesion)

Gastric MALTL^*∗*^	0	0	0	Primary gastric lesion	Stage I. Positive endoscopy

Intestinal MALTL^*∗*^	II	II	0	Primary intestinal lesion, mesenteric lymph nodes	Stage II. Positive CT scan, thickened bowel wall and mesenteric lymph nodes. Radiological and clinical follow-up (partial remission after therapy on ^18^F-FDG-PET/CT and WB-DW-MRI)

Gastric MALTL^*∗*^	II	II	0	Primary gastric lesion, mesenteric, paragastric, para-aortic, and paracaval adenopathy	Stage II. Positive endoscopy. ^18^F-FDG-PET/CT (only on CT) and WB-DW-MRI detected adenopathy but failed to find the primary lesion ^18^F-FDG-PET negative

Intestinal DLBCL^*∗∗*^	IV	III	III	Bone marrow involvement	Positive bone marrow biopsy

^*∗*^MALTL, mucosa-associated lymphoid tissue lymphoma; ^*∗∗*^DLBCL, diffuse large B-cell lymphoma.

## References

[1] Freeman C., Berg J. W., Cutler S. J. (1972). Occurrence and prognosis of extranodal lymphomas. *Cancer*.

[2] Dawson I. M., Cornes J. S., Morson B. C. (1961). Primary malignant lymphoid tumours of the intestinal tract. Report of 37 cases with a study of factors influencing prognosis. *British Journal of Surgery*.

[3] Lewin K. J., Ranchod M., Dorfman R. F. (1978). Lymphomas of the gastrointestinal tract: a study of 117 cases presenting with gastrointestinal disease. *Cancer*.

[4] Herrmann R., Panahon A. M., Barcos M. P., Walsh D., Stutzman L. (1980). Gastrointestinal involvement in non-Hodgkin's lymphoma. *Cancer*.

[5] Rizvi M. A., Evens A. M., Tallman M. S., Nelson B. P., Rosen S. T. (2006). T-cell non-Hodgkin lymphoma. *Blood*.

[6] Ansell S. M., Armitage J. (2005). Non-Hodgkin lymphoma: diagnosis and treatment. *Mayo Clinic Proceedings*.

[7] Musshoff K. (1977). Clinical staging classification of non Hodgkin's lymphomas. *Strahlentherapie*.

[8] Ruskoné-Fourmestroux A., Drogosics B., Morgner A., Wotherspoon A., De Jong D. (2003). Paris staging system for primary gastrointestinal lymphomas. *Gut*.

[9] Antoch G., Vogt F. M., Freudenberg L. S. (2003). Whole-body dual-modality PET/CT and whole-body MRI for tumor staging in oncology. *The Journal of the American Medical Association*.

[10] Ghimire P., Wu G.-Y., Zhu L. (2011). Primary gastrointestinal lymphoma. *World Journal of Gastroenterology*.

[11] Di Raimondo F., Caruso L., Bonanno G. (2007). Is endoscopic ultrasound clinically useful for follow-up of gastric lymphoma?. *Annals of Oncology*.

[12] Padhani A. R., Koh D.-M., Collins D. J. (2011). Whole-body diffusion-weighted MR imaging in cancer: current status and research directions. *Radiology*.

[13] Lin C., Luciani A., Itti E. (2010). Whole-body diffusion-weighted magnetic resonance imaging with apparent diffusion coefficient mapping for staging patients with diffuse large B-cell lymphoma. *European Radiology*.

[14] Mayerhoefer M. E., Karanikas G., Kletter K. (2014). Evaluation of diffusion-weighted MRI for pretherapeutic assessment and staging of lymphoma: results of a prospective study in 140 patients. *Clinical Cancer Research*.

[15] Takahara T., Imai Y., Yamashita T., Yasuda S., Nasu S., Van Cauteren M. (2004). Diffusion weighted whole body imaging with background body signal suppression (DWIBS): technical improvement using free breathing, STIR and high resolution 3D display. *Radiation Medicine*.

[16] Gu J., Chan T., Zhang J., Leung A. Y. H., Kwong Y.-L., Khong P.-L. (2011). Whole-body diffusion-weighted imaging: the added value to whole-body MRI at initial diagnosis of lymphoma. *American Journal of Roentgenology*.

[17] Abdulqadhr G., Molin D., Åström G. (2011). Whole-body diffusion-weighted imaging compared with FDG-PET/CT in staging of lymphoma patients. *Acta Radiologica*.

[18] Koh D.-M., Collins D. J. (2007). Diffusion-weighted MRI in the body: applications and challenges in oncology. *American Journal of Roentgenology*.

[19] Lin C., Itti E., Luciani A., Haioun C., Meignan M., Rahmouni A. (2010). Whole-body diffusion-weighted imaging in lymphoma. *Cancer Imaging*.

[20] Kellenberger C. J., Miller S. F., Khan M., Gilday D. L., Weitzman S., Babyn P. S. (2004). Initial experience with FSE STIR whole-body MR imaging for staging lymphoma in children. *European Radiology*.

[21] Kwee T. C., Takahara T., Luijten P. R., Nievelstein R. A. J. (2010). ADC measurements of lymph nodes: inter- and intra-observer reproducibility study and an overview of the literature. *European Journal of Radiology*.

[22] Pakos E. E., Fotopoulos A. D., Ioannidis J. P. A. (2005). ^18^F-FDG PET for evaluation of bone marrow infiltration in staging of lymphoma: a meta-analysis. *Journal of Nuclear Medicine*.

[23] Cheson B. D., Horning S. J., Coiffer B. (1999). Report of an international workshop to standardize response criteria for non-Hodgkin's lymphomas. NCI sponsored international working group. *Clinical Oncology*.

[24] Seam P., Juweid M. E., Cheson B. D. (2007). The role of FDG-PET scans in patients with lymphoma. *Blood*.

[25] Perry C., Herishanu Y., Metzer U. (2007). Diagnostic accuracy of PET/CT in patients with extranodal marginal zone MALT lymphoma. *European Journal of Haematology*.

[26] Brenner D. J., Elliston C. D. (2004). Estimated radiation on risks potentially associated with full-body CT screening. *Radiology*.

[27] Huang B., Law M. W.-M., Khong P.-L. (2009). Whole-body PET/CT scanning: estimation of radiation dose and cancer risk. *Radiology*.

[28] Plathow C., Walz M., Lichy M. P. (2008). Cost considerations for whole-body MRI and PET/CT as part of oncologic staging. *Der Radiologe*.

[29] Wotherspoon A. C., Doglioni C., Isaacson P. G. (1992). Low-grade gastric B-cell lymphoma of mucosa-associated lymphoid tissue (MALT): a multifocal disease. *Histopathology*.

[30] Cohen S. M., Petryk M., Varma M., Kozuch P. S., Ames E. D., Grossbard M. L. (2006). Non-Hodgkin's lymphoma of mucosa-associated lymphoid tissue. *Oncologist*.

[31] Deutsch A. J. A., Aigelsreiter A., Staber P. B. (2007). MALT lymphoma and extranodal diffuse large B-cell lymphoma are targeted by aberrant somatic hypermutation. *Blood*.

[32] Boot H. (2010). Diagnosis and staging in gastrointestinal lymphoma. *Best Practice and Research: Clinical Gastroenterology*.

[33] Cheson B. D., Pfistner B., Juweid M. E. (2007). Revised response criteria for malignant lymphoma. *Journal of Clinical Oncology*.

